# A Phosphoproteomic Approach towards the Understanding of the Role of TGF-β in *Trypanosoma cruzi* Biology

**DOI:** 10.1371/journal.pone.0038736

**Published:** 2012-06-12

**Authors:** Patrícia M. Ferrão, Fabiane L. de Oliveira, Wim M. Degrave, Tania C. Araujo-Jorge, Leila Mendonça-Lima, Mariana C. Waghabi

**Affiliations:** 1 Laboratório de Genômica Funcional e Bioinformática, Instituto Oswaldo Cruz, Fundação Oswaldo Cruz, Rio de Janeiro, Brazil; 2 Laboratório de Inovações em Terapias, Ensino e Bioprodutos, Instituto Oswaldo Cruz, Fundação Oswaldo Cruz, Rio de Janeiro, Brazil; Federal University of São Paulo, Brazil

## Abstract

Transforming growth factor beta (TGF-β) plays a pivotal role in Chagas disease, not only in the development of chagasic cardiomyopathy, but also in many stages of the *T. cruzi* life cycle and survival in the host cell environment. The intracellular signaling pathways utilized by *T. cruzi* to regulate these mechanisms remain unknown. To identify parasite proteins involved in the TGF-β response, we utilized a combined approach of two-dimensional gel electrophoresis (2DE) analysis and mass spectrometry (MS) protein identification. Signaling via TGF-β is dependent on events of phosphorylation, which is one of the most relevant and ubiquitous post-translational modifications for the regulation of gene expression, and especially in trypanosomatids, since they lack several transcriptional control mechanisms. Here we show a kinetic view of *T. cruzi* epimastigotes (Y strain) incubated with TGF-β for 1, 5, 30 and 60 minutes, which promoted a remodeling of the parasite phosphorylation network and protein expression pattern. The altered molecules are involved in a variety of cellular processes, such as proteolysis, metabolism, heat shock response, cytoskeleton arrangement, oxidative stress regulation, translation and signal transduction. A total of 75 protein spots were up- or down-regulated more than twofold after TGF-β treatment, and from these, 42 were identified by mass spectrometry, including cruzipain–the major *T. cruzi* papain-like cysteine proteinase that plays an important role in invasion and participates in the escape mechanisms used by the parasite to evade the host immune system. In our study, we observed that TGF-β addition favored epimastigote proliferation, corroborating 2DE data in which proteins previously described to be involved in this process were positively stimulated by TGF-β.

## Introduction

*Trypanosoma cruzi* (*T. cruzi*) is a flagellate parasite that causes Chagas disease - a widely distributed, debilitating illness that constitutes a serious health problem in Latin America, affecting 10–12 million people and killing over 15,000 each year [1]. In this disease, transforming growth factor beta (TGF-β) seems to play a pivotal role in parasite infection, multiplication and the degree and rate of cardiac fibrosis. TGF-β is involved in acute and chronic chagasic cardiopathy. High levels of TGF-β and the activation of its signaling pathway were shown to be peculiar aspects in patients with chronic Chagas disease [2], [3]. We recently demonstrated a beneficial action of a TGF-β signalling inhibitor (SB-431542) administered during the acute phase of experimental Chagas disease, indicating that inhibition of TGF-β-induced activity could represent a new therapeutic action for acute and chronic Chagas disease treatment [4].

Besides its relevant role in the pathology of Chagas disease, TGF-β was also observed to be intimately associated as a regulator of different stages of the *T. cruzi* life cycle: 1) TGF-β can promote parasite survival [4]; 2) host cell infection by *T. cruzi* is dependent on active TGF-β and requires fully functional TGF-β receptors [5]–[8]; 3) *T. cruzi* infection is able to induce the expression of TGF-β in different models [2], [9]–[11]; 4) the parasites are able to directly activate latent TGF-β [7] and 5) amastigote forms of *T. cruzi*, once in the cytoplasm, internalize host cell TGF-β, thereby regulating their own intracellular life cycle [12]. Taken together, these data indicate an important role for TGF-β in *T. cruzi* biology. However, the mechanisms used by the parasite to recognize and respond to this host-derived factor remain partially unknown.

TGF-β belongs to a group of structurally related polypeptides collectively called “TGF-β superfamily”. Members of this family are involved in the regulation of a large variety of processes, such as cell growth, tissue remodeling, development, differentiation, motility, angiogenesis, inflammation, immune regulation, fibrosis, apoptosis and tumorigenesis [13]–[16]. In its classic pathway, TGF-β signaling begins by ligand binding to a transmembrane receptor with intracellular serine/threonine kinase activity, known as TGF-β receptor-II (TRII). Upon ligand binding, TRII phosphorylates and stimulates the serine/threonine kinase activity of TGF-β receptor-I (TRI). Once activated, TRI phosphorylates the cytoplasmic signaling proteins Smad-2 and Smad-3, which then associate with Smad-4, translocate into the nucleus as a multiprotein complex, and stimulate the transcription of TGF-β responsive genes [17]. Several non-Smad signaling pathways are also known to be activated or modulated by TGF-β in eukaryotic cells. These include Jun-kinase, p38 MAP kinase, Ras/MEK/ERK, Rho-A/p160ROCK, and PP2A/S6 kinase, which are known as alternative pathways induced by TGF-β [18]. Interestingly, homologs of Ras [19] Rho [20] and ERK [21], [22] have been characterized in *T. cruzi* and *T. brucei*, suggesting that at least some of these alternative TGF-β pathways might be functional in these parasites. Overall, signaling via TGF-β seems always to be dependent on phosphorylation, which is one of the most relevant and ubiquitous post-translational modifications (PTM). This PTM is related to many molecular mechanisms, such as protein activation, localization, interaction and turnover. Phosphorylation is regulated by a highly dynamic network of protein kinases and phosphatases that modulate crucial cellular functions, including cell growth, proliferation, differentiation, migration, metabolism and apoptosis [23], [24].

The identification of differential patterns of protein phosphorylation is possible through the use of a phosphoproteomic approach, which provides insights into signal transduction pathways, triggered by growth factors, including TGF-β [25], [26]. Proteomic technology has already been used to study the three most clinically relevant trypanosomatids *T. cruzi*, *Leishmania* and *T. brucei* [23], [27]–[34], performing as a powerful tool for the study of global gene expression patterns, since it enables the analysis of the whole protein profile of an organism in a single experiment. It is worth reminding that trypanosomatids indiscriminately transcribe most genes in large polycistronic units [35], [36], unlike metazoa and yeast, which use regulated transcription factors to direct the expression of certain genes, thus emphasizing the critical role of PTM in the regulation of *T. cruzi* proteins.

Due to the relevant role of TGF-β in *T. cruzi* biology, a study of the proteins involved in TGF-β response is of crucial interest to understand how a host molecule can interfere in the parasite’s developmental process. Therefore, the aim of the present study is to characterize *T. cruzi* molecules responsive to TGF-β through a combined approach employing two-dimensional gel electrophoresis (2DE) analysis and mass spectrometry (MS) protein identification. Here we show that incubation of *T. cruzi* epimastigotes (Y strain) with TGF-β promotes a remodeling of the parasite phosphorylation network and protein expression pattern influencing cellular processes such as metabolism, heat shock response, cytoskeleton arrangement, oxidative stress regulation, translation, signal transduction and proteolysis. We also observed that TGF-β stimulated many biological events in different forms of *T. cruzi*, such as epimastigote proliferation and differentiation of trypomastigotes into amastigotes (amastigogenesis).

## Materials and Methods

### Cell Culture

Epimastigote forms of *T. cruzi* (Y strain) were grown in liver infusion tryptose (LIT) medium supplemented with 10% fetal bovine serum at 28°C.

### Sample Preparation

Epimastigotes (5×10^8^) from 5-day-old cultures (exponential growth phase) were centrifuged and washed two times with phosphate buffered saline containing bovine serum albumin (PBS/BSA 0.1%) and resuspended in the same buffer. Parasites were incubated with TGF-β (5 ng/ml) for 1, 5, 30 or 60 minutes. The samples were then centrifuged and washed twice with PBS, resuspended in lysis buffer (PBS containing 1∶100 protease and phosphatase inhibitor cocktails from Sigma) and submitted to four cycles of freeze–thawing. Samples were precipitated in 17% tricholoracetic acid, centrifuged and the pellet washed in cold acetone/triethanolamine 1%. The samples were solubilized in isoelectric focusing buffer (2% CHAPS, 8 M urea) containing phosphatase inhibitor cocktail (Sigma) and stored at −70°C. Protein concentration was determined by the RCDC method (BioRad), using bovine serum albumin as standard.

### Two-Dimensional Electrophoresis (2DE)

Nonlinear IPG strips in the pH range 3–10 were rehydrated in a buffer (8 M urea, 2 mM tributhylphosphine, 1% ampholytes, 2% CHAPS) containing 100 µg (7 cm) or 500 µg (17 cm) of total protein extracts. Isoelectric focusing was conducted on a Protein IEF Cell (BioRad) according to the manufacturer’s instructions. The strips were then re-equilibrated sequentially with 130 mM DTT and 135 mM iodoacetamide in equilibration buffer (6 M urea, 20% glycerol, 2% SDS) for 15 minutes each. Proteins within the equilibrated strips were resolved on 12% SDS-PAGE gels.

### Gel Staining

Gels were stained sequentially with phosphoprotein-specific Pro-Q Diamond stain and the total protein stain Sypro Ruby (both from Molecular Probes, Invitrogen) according to the manufacturer’s protocols. After that, gels were stained with colloidal Coomassie Brilliant Blue G-250 (BioRad) as described elsewhere [37].

### Image Analysis

Protein spots were visualized by scanning the stained gels using proper excitation and emission wavelengths in a Typhoon Trio scanner (Applied Biosystems) and the obtained images were analyzed with the PDQuest software version 8.0.1 (BioRad). The spots were quantified on the basis of their relative volume: the amount of a protein spot was expressed as the sum of the intensities of all the pixels that made up the spot. As positive and negative controls for the staining of phosphorylated proteins we used the PeppermintStick™ phosphoprotein molecular weight standard (Molecular Probes, Invitrogen), which is composed of a mixture of phosphorylated and non-phosphorylated proteins. To ensure that the values found were due to protein phosphorylation and not to a higher load of proteins in acrylamide gels, we used a ratiometric parameter by dividing the value obtained in Pro-Q staining by the value obtained in Sypro Ruby staining for each spot of interest. In this study, the analyses were based on the comparison of protein expression and/or phosphorylation from *T. cruzi* epimastigote samples treated or not with TGF-β at different times (1, 5, 30 or 60 minutes). Spots displaying at least a two-fold difference in their pixel intensity were assigned as differentially expressed and/or phosphorylated. We analyzed three to four gels for each experimental condition and selected only spots with normalized volumes that showed more than 2-fold increase or decrease after TGF-β stimulation in at least one of the studied time points.

**Figure 1:**
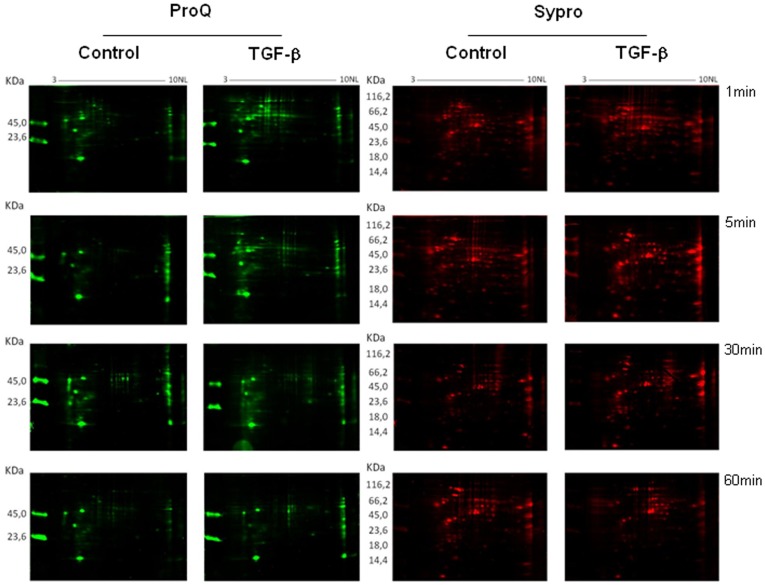
Bidimensional phosphoproteome of *T. cruzi* Y strain epimastigotes. Representative images show the total protein patterns of *T. cruzi* treated or not with TGF-β for 1, 5, 30 or 60 minutes. Protein extracts (100 µg) were separated on 7 cm pH 3–10NL IPG strips and 12% SDS-PAGE gels. Phosphoproteins were stained with Pro-Q Diamond (shown in green) and total protein stained with Sypro Ruby (shown in red). Images were artificially colored using PD Quest (BioRad) software tools. Phosphoprotein staining was confirmed with the visualization of the two positive molecular weight control bands (Peppermint Stick – Molecular Probes). The molecular weight (MW) of marker proteins and the pH range of the IEF gradient are indicated. N=3.

**Table 1:** TGF-β responsive proteins identified by MALDI TOF-TOF.

Spot n°	Identification	Accession Number	pI theor	pI exp	MW theor (KDa)	MW exp (KDa)	Score	Sequence Coverage (%)	N° of Identified Peptides
**HEAT SHOCK PROTEINS**									
1	DnaK	gi|71649573	4,88	4,7	30,1	83,5	87	10%	2
2	HSP70	gi|71406304	6,19	5,2	40,8	84	446	24%	8
3	HSP70	gi|71406304	6,19	5,3	40,8	83,6	450	24%	8
4	HSP70	gi|123603	5,44	5,4	73,7	83,2	123	8%	4
5	Mitochondrial precursor HSP70	gi|71407515	5,75	5,4	70,9	80,5	198	7%	4
6	Mitochondrial precursor HSP70	gi|71407515	5,75	5,6	70,9	81	154	8%	4
7	Mitochondrial precursor HSP60	gi|71665064	5,73	5	59,3	74,6	258	13%	5
8	Mitochondrial precursor HSP60	gi|71665068	5,73	5,1	59,3	74,5	345	17%	7
14	HSC70	gi|71663660	4,91	4.2	41,5	58,8	159	10%	4
37	co-chaperone GrpE	gi|71401098	8,49	6,2	24,3	19,8	89	11%	2
42	Cyclophilin A	gi|71659715	8,44	8,9	18,7	14	209	27%	4
**CYTOSKELETON PROTEINS**									
9	beta-tubulin	gi|18568139	4,74	4,4	49,6	66,4	60	4%	2
27	beta-tubulin	gi|1220547	4,69	4,7	49,4	38,5	266	10%	4
**METABOLISM PROTEINS**									
11	Prostaglandin F2 alpha synthase	gi|945108	6,03	6	42,2	67,8	81	3%	1
12	Prostaglandin F2 alpha synthase	gi|945108	6,03	6,2	42,2	67,5	358	18%	6
20	Prostaglandin F2 alpha synthase	gi|71659766	6,03	6,2	42,2	67,5	357	18%	6
21	Prostaglandin F2 alpha synthase	gi|945108	6,03	6,1	42,2	48,6	285	18%	6
22	Prostaglandin F2 alpha synthase	gi|945108	6,03	6,3	42,2	48,6	415	18%	7
23	Prostaglandin F2 alpha synthase	gi|25006239	5,83	6,4	42,2	48,6	203	9%	3
16	Enolase	gi|5566209	5,59	6,6	37,6	58	118	9%	3
17	Enolase	gi|71665461	5,92	6,6	46,4	58	173	13%	4
18	Alanine aminotransferase	gi|71667866	7.48	7,5	54,8	58,8	40	2%	1
19	Tyrosine aminotransferase	gi|71659493	5,82	6,3	46,1	51	122	10%	4
26	Protein disulfide isomerase	gi|71410849	7,79	7,1	29,5	46,3	76	5%	1
29	Aromatic L-alpha-hydroxyacid dehydrogenase	gi|7109725	6,82	7	33,7	41,5	67	9%	2
31	Aldo-keto reductase	gi|189396135	7,18	7,9	32,4	31,1	177	13%	5
40	GAPDH	gi|120679	8,87	9,5	39	45,1	79	4%	1
41	Glycosomal malate dehydrogenase	gi|71423452	8,82	9,6	34,1	37,4	42	3%	1
**HYPOTHETICAL PROTEINS**									
13	Hypothetical protein	gi|71661816	6,17	6,4	54,7	67,6	99	10%	4
24	Hypothetical protein	gi|71405542	4,86	3,9	93	43,6	54	2%	2
25	Hypothetical protein	gi|71409780	4,85	3,9	93,6	40,5	69	1%	2
**TRANSLATION-ASSOCIATED PROTEINS**									
28	Elongation factor 1 alpha	gi|704459	7,55	6,6	43,5	41,9	105	4%	2
30	Nascent polypeptide associated complex subunit	gi|71419111	4,66	4,1	19,5	30,6	167	17%	2
35	Proteasome alpha 1 subunit	gi|71415419	5,45	5,7	29,3	22,5	35	5%	1
36	Eukaryotic initiation factor 5a	gi|71659667	4,82	4,2	18	15,7	394	35%	4
39	Elongation factor 1 alpha	gi|704459	7,55	9,6	43,5	61	123	18%	4
**PROTEASES/PEPTIDASES**									
10	peptidase M20/M25/M40	gi|71421293	5,29	5,1	51,1	59,8	49	2%	1
		gi|71649847	5,35		51,2		85	4%	2
15	Cruzipain	gi|1136308	5,63	3,9	49,6	53,3	106	2%	3
33	hslvu complex proteolytic subunit-like	gi|71416273	6,77	5,2	22,8	22,8	157	15%	3
**OXIDATIVE STRESS REGULATING PROTEINS**									
38	Tryparedoxin peroxidase	gi|17224953	5,96	6,8	22,2	21,8	77	12%	3
**SIGNAL TRANSDUCTION PROTEINS**									
32	Methylthioadenosine phosphorylase	gi|71655964	6,34	8,1	33,1	26,7	72	10%	2
34	IgE-dependent histamine releasing factor	gi|71407834	4,52	4.0	19,5	18,7	184	25%	3

pI theor = theoretical isoelectric point.

pI exp = experimental isoelectric point.

MW theor = theoretical molecular weight.

MW exp = experimental molecular weight.

**Figure 2:**
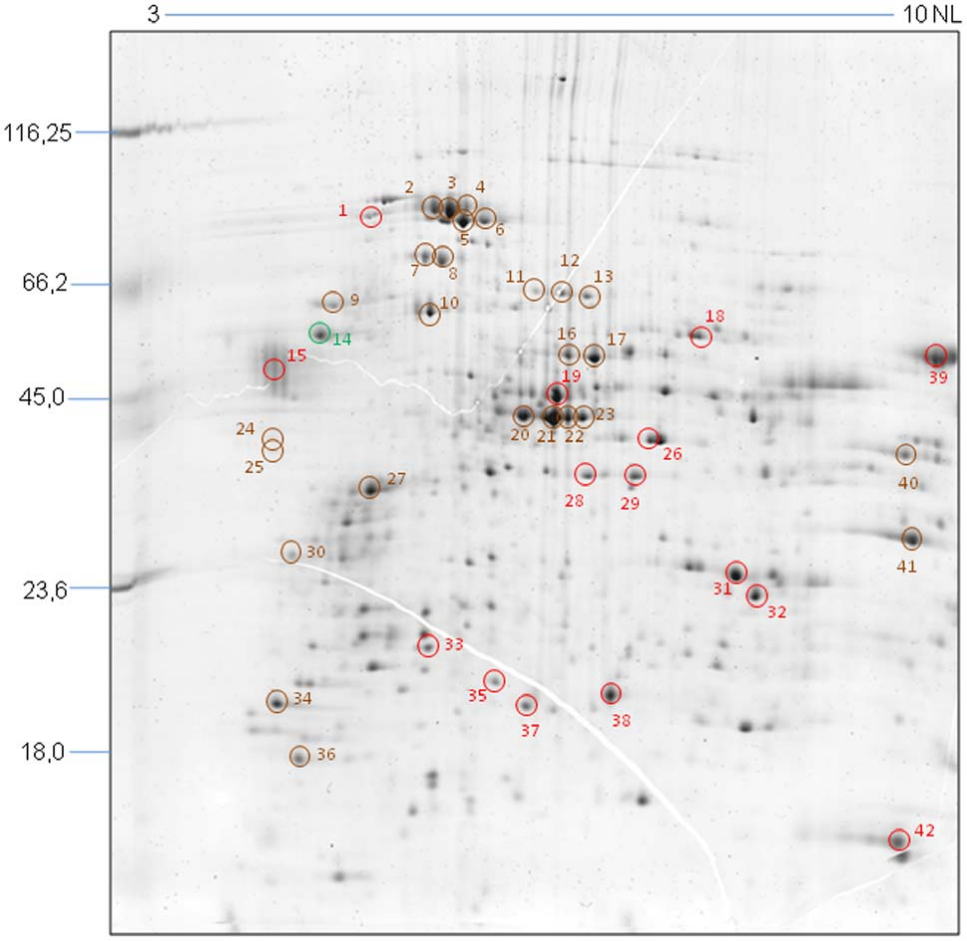
Proteomic map of *T. cruzi* epimastigotes. The image shows the total protein pattern of epimastigotes treated with TGF-β for 1 minute and separated by 2D-PAGE (17 cm IPG strips in the pH range 3-10NL and 12% SDS-PAGE, CBB-G250 staining). The colored circles indicate protein spots identified by mass spectrometry that differ significantly only in their phosphorylation (green), or in their expression (red) or in both (brown) levels in one or more studied time points. The molecular weight (MW) of marker proteins and the pH range of the IEF gradient are indicated. N=3.

**Table 2:** Proteins with changes in phosphorylation pattern in response to TGF-β.

			Fold Changes			
FUNCTIONAL GROUPS	SPOT NUMBER	PROTEIN NAME	1 min	5 min	30 min	60 min
**HEAT SHOCK PROTEINS**	5	mitochondrial precursor HSP70	8,39	0,72	1,76	0,43
	6	mitochondrial precursor HSP70	3,22	0,00*	4,23	2,43
	3	HSP70	1,95	1,09	2,05	1,48
	7	mitochondrial precursor HSP60	3,65	0,00*	1,11	1,92
	14	Hsc70	1,90	1,33	1,35	0,55
**CYTOSKELETON PROTEINS**	9	beta-tubulin	1,06	2,88	2,13	0,49
	27	beta-tubulin	3,13	1,00	1,33	0,53
**METABOLISM PROTEINS**	11	Prostaglandin F2 alpha synthase	6,36	0,00*	0,00*	3,91
	12	Prostaglandin F2 alpha synthase	5,10	4,48	0,82	3,72
	21	Prostaglandin F2 alpha synthase	0,45	0,24	3,34	0,37
	16	Enolase	3,26	0,08	0,31	1,61
	17	Enolase	0,00*	0,00*	2,06	2,30
	40	GAPDH	3,42	0,43	1,13	2,52
	41	Glycosomal malate dehydrogenase	0,35	2,12	1,66	24,07
**HYPOTHETICAL PROTEINS**	24	Hypothetical protein	1,79	0,35	0,16	0,00*
	25	Hypothetical protein	0,63	0,58	2,05	4,28
**TRANSLATION-ASSOCIATED PROTEINS**	30	Nascent polypeptide associated complex subunit	2,31	0,47	3,99	0,53
	36	Eukaryotic initiation factor 5a	0,62	0,93	0,70	2,19
**SIGNAL TRANSDUCTION PROTEINS**	34	IgE-dependent histamine-releasing factor	1,90	0,69	4,58	18,58
**PROTEASES/PEPTIDASES**	10	peptidase M20/M25/M40	4,39	0,00*	2,48	2,00

Fold changes presents the ratio compared with values in the absence of TGF-β stimulation.

* Values equal to zero correspond to spots that could not be detected on gels.

**Figure 3:**
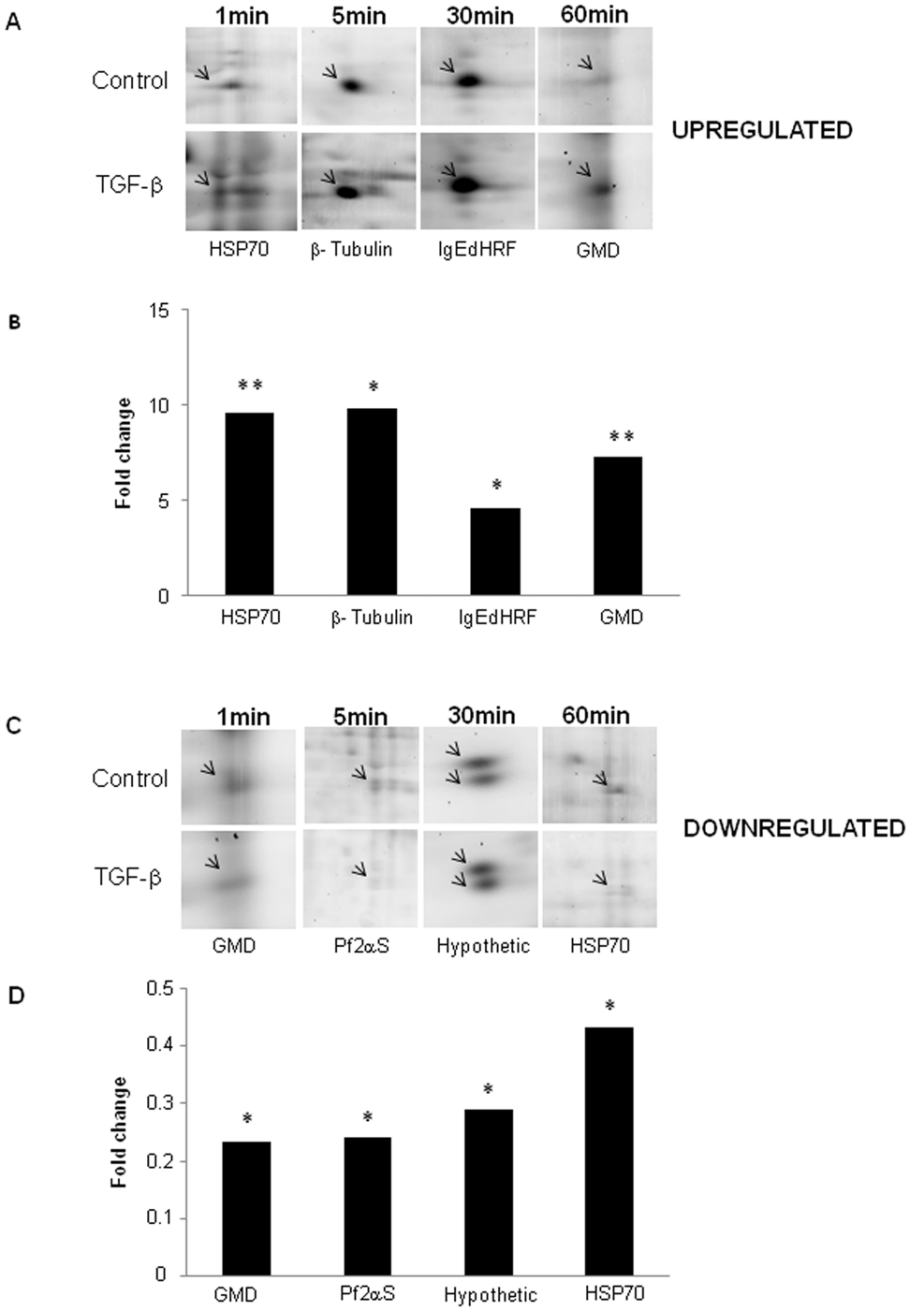
Proteins that presented the major phosphorylation changes in response to TGF-β. Magnified regions showing protein spots that presented the greatest up- (A) or down-(C) phosphoregulation and their respective bar graphics (B and D) with the mean fold change values.

**Table 3:** Proteins with changes in expression pattern in response to TGF-β.

			Fold Changes			
FUNCTIONAL GROUPS	SPOT NUMBER	PROTEIN NAME	1 min	5 min	30 min	60 min
**HEAT SHOCK PROTEINS**	5	mitochondrial precursor Hsp70	1,02	1,01	0,64	0,65
	6	mitochondrial precursor Hsp70	1,50	1,58	4,23	2,43
	4	Hsp70	1,04	6,15	0,54	0,41
	3	Hsp70	0,00*	1,55	0,41	0,05
	7	mitochondrial precursor Hsp60	1,62	0,85	0,32	0,68
	8	mitochondrial precursor Hsp60	1,09	1,28	0,67	0,30
	1	DnaK	3,90	3,13	0,00*	0,00*
	14	Hsc70	1,08	1,55	1,14	1,22
	37	GrpE	0,61	0,93	2,83	2,24
	42	Cyclophilin A	5,45	1,11	4,13	0,63
**CYTOSKELETON PROTEINS**	9	beta-tubulin	2,23	3,39	0,38	0,31
	27	beta-tubulin	1,10	1,27	0,62	0,49
**METABOLISM PROTEINS**	11	Prostaglandin F2 alpha synthase	1,18	1,70	1,16	0,67
	12	Prostaglandin F2 alpha synthase	0,26	0,84	0,95	0,73
	20	Prostaglandin F2 alpha synthase	1,51	1,00	0,22	0,53
	21	Prostaglandin F2 alpha synthase	1,37	4,14	0,27	1,67
	22	Prostaglandin F2 alpha synthase	2,58	2,95	0,26	1,18
	23	Prostaglandin F2 alpha synthase	0,65	3,52	6,20	0,50
	31	Aldo-ketoreductase	0,81	1,29	0,38	0,79
	16	Enolase	0,58	13,06	1,48	1,32
	17	Enolase	0,80	0,60	0,18	1,92
	18	Alanine aminotransferase	1,32	8,05	1,27	5,64
	40	GAPDH	0,50	2,34	0,95	0,55
	41	Glycosomal malate dehydrogenase	1,06	3,37	1,37	0,24
	26	Protein disulfide isomerase	1,09	8,07	1,05	1,32
	29	Aromatic L-alpha-hydroxyacid dehydrogenase	0,99	2,70	0,42	0,97
	19	Tyrosine aminotransferase	1,12	1,38	1,69	1,19
**HYPOTHETICAL PROTEINS**	24	Hypothetical protein	1,17	0,73	16,71	1,29
	25	Hypothetical protein	0,56	7,84	1,58	0,33
	13	Hypothetical protein	2,20	8,03	0,32	7,24
**TRANSLATION-ASSOCIATED** **PROTEINS**	30	Nascent polypeptide associated complex subunit	1,07	1,57	1,38	1,04
	36	Eukaryotic initiation factor 5a	1,46	0,60	0,95	1,03
	28	Elongation factor 1 alpha	0,95	24,62	0,54	1,24
	39	Elongation factor 1 alpha	1,09	4,20	1,55	0,29
	35	Proteasome alpha 1 subunit	0,81	1,42	0,86	2,80
**SIGNAL TRANSDUCTION PROTEINS**	32	Methylthioadenosine phosphorylase	0,55	0,36	0,72	0,73
	34	IgE-dependent histamine-releasing factor	0,80	0,70	1,82	0,26
**PROTEASES/PEPTIDASES**	15	Cruzipain	0,65	38,07	0,42	1,49
	10	peptidase M20/M25/M40	1,62	10,03	1,14	0,45
	33	hslvu complex proteolytic subunit-like	0,94	4,00	0,79	0,59
**OXIDATIVE STRESS REGULATING PROTEINS**	38	Tryparedoxin peroxidase	0,55	1,38	0,71	11,82

Fold changes presents the ratio compared with values in the absence of TGF-β stimulation.

* Values equal to zero correspond to spots that could not be detected on gels.

**Figure 4:**
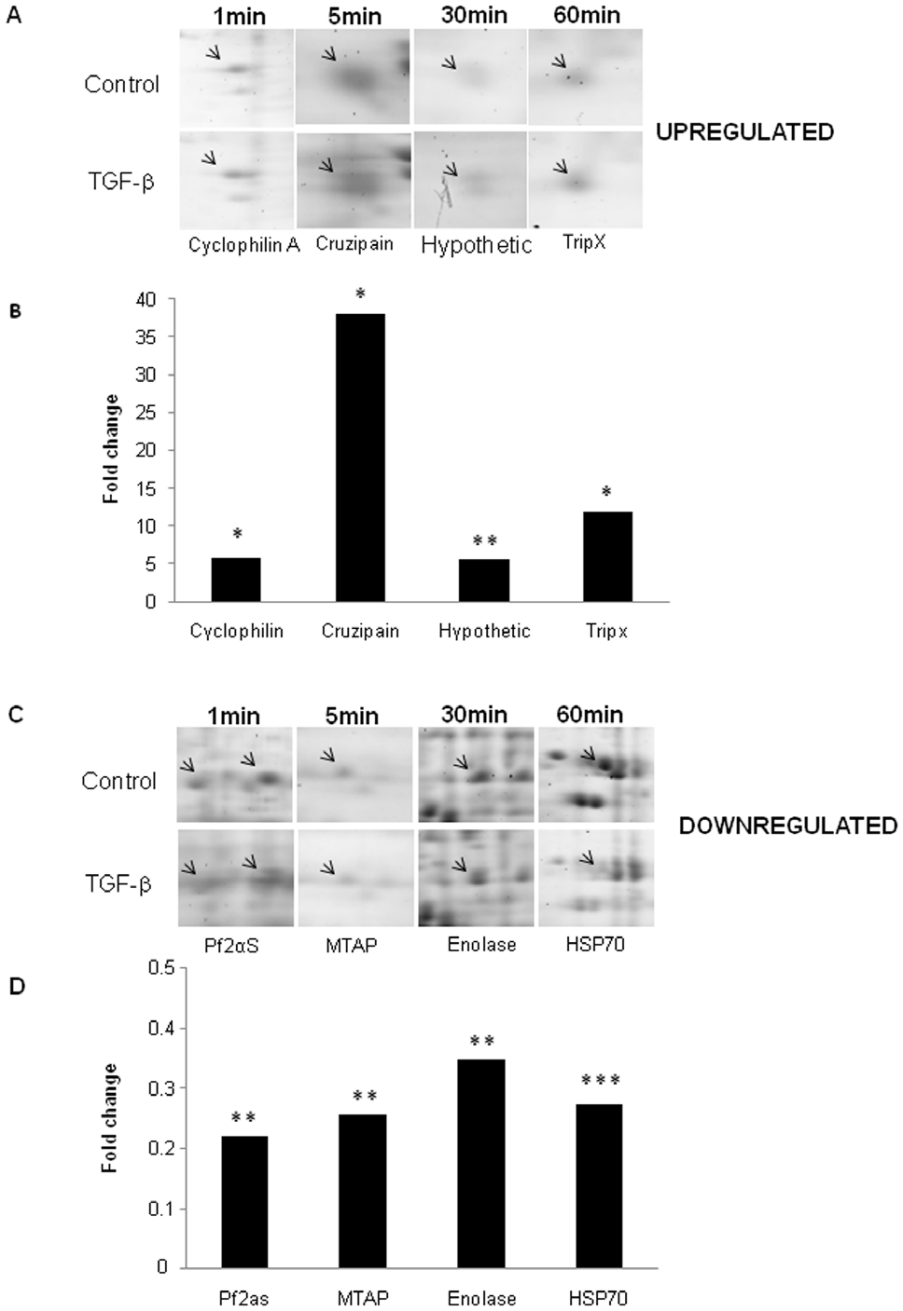
Proteins that presented the major expression changes in response to TGF-β. Magnified regions showing protein spots that presented the greatest up- (A) or down-(C) regulation in expression and their respective bar graphics (B and D) with the mean fold change values.

**Figure 5:**
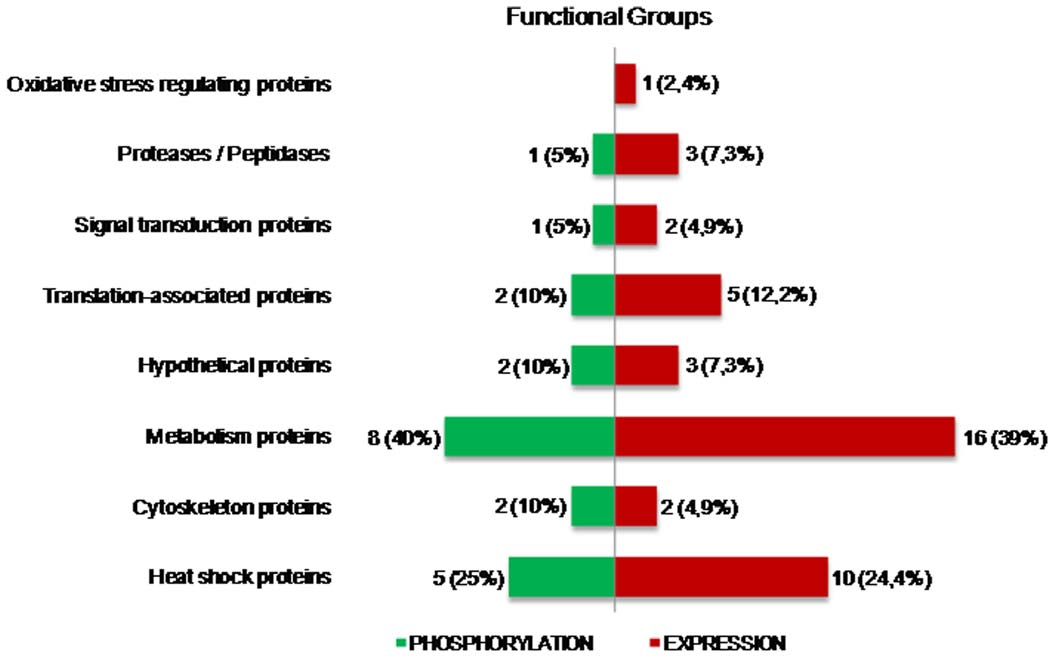
Distribution of the identified proteins into functional groups. Bar graphs presenting the functional groups of proteins that had their expression (red) or phosphorylation (green) regulated by TGF-β.

**Figure 6:**
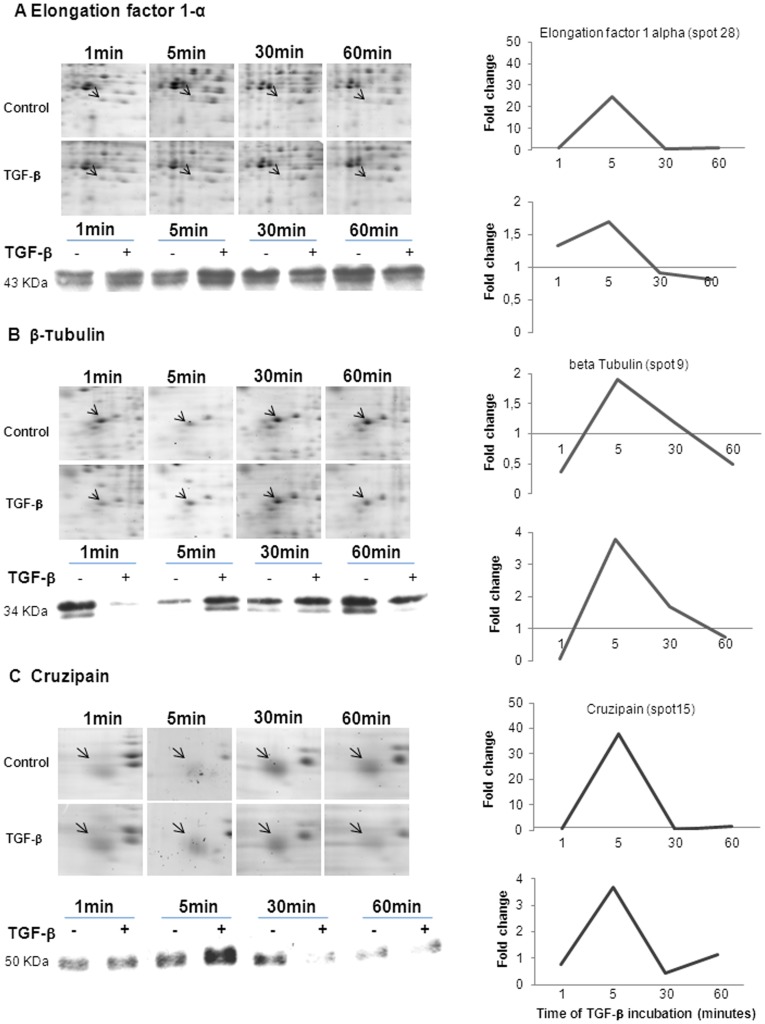
Kinetic view of protein expression in response to TGF-β. Expression pattern of elongation factor 1-α (A), β-tubulin (B) and cruzipain (C) from *T. cruzi* epimastigotes incubated with TGF-β at different periods of time. The graphics represent the values found for the kinetic of these proteins obtained through a 2DE approach and Western blot analysis.

**Figure 7:**
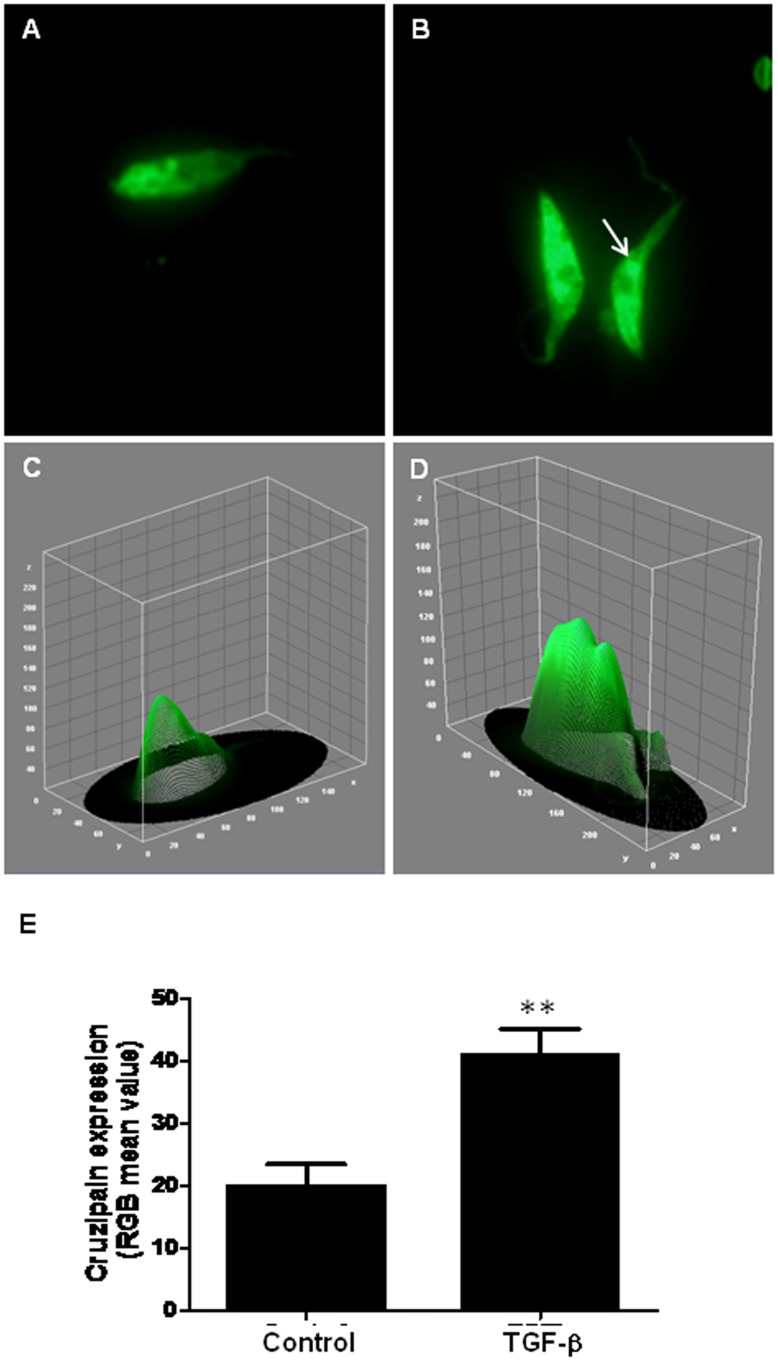
TGF-β induces cruzipain expression in *T. cruzi* epimastigotes. Parasites treated (B) or not with TGF-β (A) for 5 minutes were immunostained for cruzipain and corresponding histograms were obtained using the ImageJ software (C and D). The quantification of the parasite areas labeled for cruzipain were determinate by the mean of RGB color and demonstrated as a bar graphic (E).

**Figure 8:**
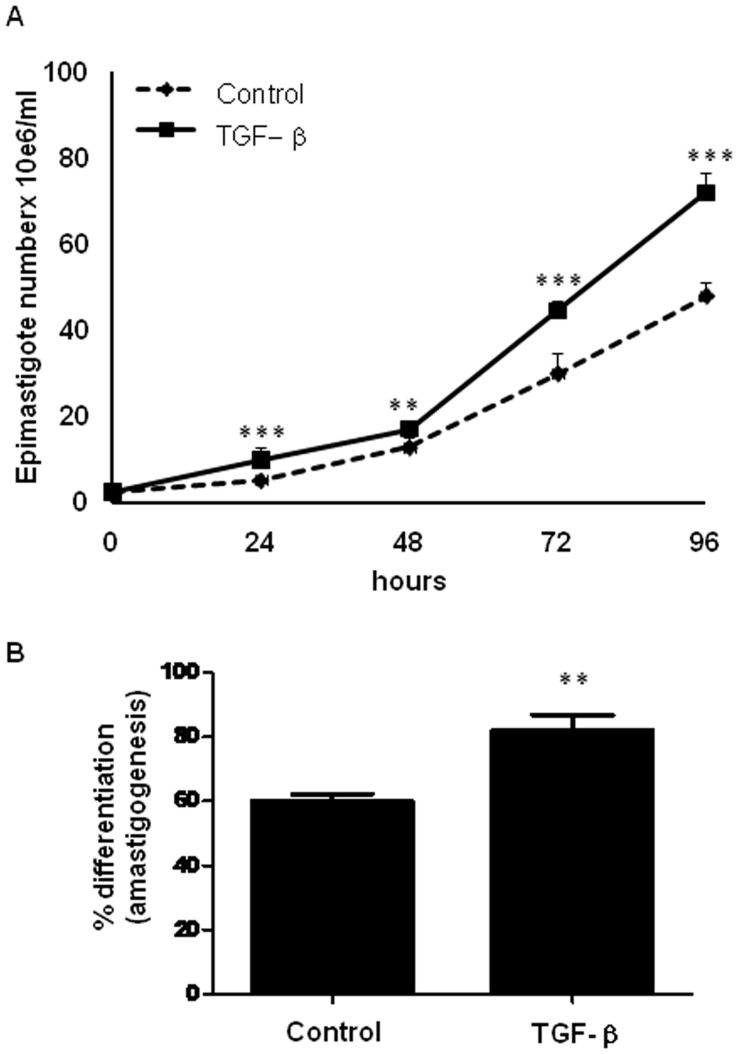
Biological effects exerted by TGF-β over different forms of *T. cruzi*. A. *In vitro* proliferation of epimastigotes. Epimastigotes were grown in the absence or presence of TGF-β (5 ng/ml, daily added) in LIT medium. The graph represents the mean number of epimastigotes present in cultures treated or not with TGF-β after 24, 48, 72 and 96 hours. N=4. **p<0.01, ***p<0.001. B. Amastigogenesis *in vitro*. Differentiation of trypomastigotes was performed by acid induction followed or not by treatment with 5 ng/ml of recombinant TGF-β 1. The graph represents the percentage of differentiation into amastigote forms after 4 hours of acid induction with or without TGF-β stimuli. N=4. **p<0.01.

### Protein Digestion and MS/MS Analysis

Selected protein spots were manually excised from 17 cm gels and placed in 0.5 ml microtubes. Protein digestion and peptide extraction were conducted as previously described [38].

### Database Searching and Criteria for Protein Identification

The Mascot software (www.matrixscience.com) was set up to search the NCBInr database assuming the digestion enzyme as trypsin and a peptide tolerance +/−1.2 Da. The following variable modifications were specified: Acetyl (protein N-term), Carbamidomethyl (C), Deamidated (NQ), Gln →Pyro-Glu (N-termQ), Glu →Pyro-Glu (N-term E), Oxidation (HW), Oxidation (M), Phospho (ST) and Phospho (Y). Criteria for a positive protein identification included Mascot scores, sequence coverage and concordance between predicted molecular mass and isoelectric point with values calculated from the 2DE gels.

### Uni-Dimensional Electrophoresis (1DE) and Western Blotting

Total protein extracts (20 µg) were mixed with protein loading buffer (62,5 mM Tris-Cl pH 6,8/SDS 3%/Glicerol 10%/β-mercaptoethanol 1∶20) to a final volume of 20 µl. The mixture was heated at 100°C for 5 minutes. The proteins were then resolved on 12% SDS-PAGE and transferred to nitrocellulose membranes (Hybond C, GE). Uniform sample loading and transfer were verified using Memcode reversible protein stain (Pierce). Non-specific binding sites were blocked by incubating the membranes with 5% (w/v) nonfat milk/TBS/Tween-20 0.1% for 1 hour at room temperature. The membranes were probed with specific primary antibodies (1∶500 rabbit anti-elongation factor 1α, 1∶20.000 mouse anti-beta-tubulin, both from Sigma-Aldrich, and 1∶10.000 rabbit anti-cruzipain, gently given by Dr. Claudia Levy) in 5% w/v nonfat milk/TBS/Tween-20 0,1% and detected with a secondary antibody conjugated to peroxidase (HRP) from Pierce for 1 hour at room temperature. Blots were developed using Supersignal West Pico Chemiluminescent Substrate (Pierce), recorded on autoradiography film and scanned with a GS-800 scanner (BioRad) at 600 dpi resolution.

### Immunocytochemical Staining

Epimastigote forms of *T. cruzi* were washed with PBS and then fixed on a 4% PFA solution for 20 min at 4°C. Fixed parasites (5×10^6^) were incubated with 0.1% Triton X-100 in PBS, followed by three 10 min blockages in PBS/2% BSA at room temperature. Then, cells were incubated with primary antibody raised against recombinant cruzipain (1∶1000) or with non-immune rabbit serum diluted 1∶100 in PBS, for 60 minutes at room temperature. After washes with PBS, parasites were incubated for 60 minutes at room temperature with secondary antibody (goat anti-rabbit Alexa 488 diluted 1∶500; Invitrogen). To stain DNA, cells were incubated with DAPI (1∶5000; Sigma). The slides were then mounted in BacLight Mounting Oil (Molecular Probes, Invitrogen) and observed under an epifluorescence microscope (Nikon). Image processing was performed using ImagePro.

### *In vitro* Proliferation of Epimastigotes

Epimastigote forms of *T. cruzi* (Y strain) were incubated with 5 ng/ml of recombinant TGF-β1 (R&D). Epimastigote proliferation rates were evaluated after 24, 48, 72 and 96 hours of TGF-β stimulation by quantifying the live parasites in a Neubauer improved counting chamber.

### *In vitro* Amastigogenesis

Infective trypomastigote forms of *T. cruzi* (Y strain) were obtained from blood of infected mice at the peak of parasitemia. In all assays, the living parasites were incubated in serum-free medium. Amastigogenesis was promoted by acid induction as previously described [39] and 5 ng/ml of recombinant TGF-β1 (R&D) were added to stimulate parasite differentiation. After 4 hours of acid induction with or without TGF-β stimuli, differentiated amastigotes were quantified in a Neubauer improved counting chamber and the percentage of differentiation determined.

## Results

### Two-dimensional Proteomic Profile of *T. cruzi* Epimastigotes in Response to TGF-β

To identify changes in protein phosphorylation and expression patterns in response to TGF-β, we compared the intracellular proteome of *T. cruzi* epimastigotes treated or not with this cytokine. In order to assess early and long-term changes in protein phosphorylation and expression patterns, cells were treated with TGF-β for 1, 5, 30 or 60 minutes and compared with non-treated cells (Figure 1).

Approximately 200 protein spots were resolved in gels stained with Sypro Ruby. A total of 75 protein spots (37.5%) were found to be more than 2-fold up- or down-regulated after TGF-β treatment. From these, 42 were identified by mass spectrometry (Table 1) and are indicated in the proteomic map shown in Figure 2.

### Protein Phosphorylation Changes in Response to TGF-β

In our analysis, 20 protein spots showed consistent phosphorylation changes during all studied times (Table 2). Phosphorylation of 50% of the identified proteins was rapidly increased after 1 minute treatment with TGF-β. Proteins with highest phosphorylation changes in response to TGF-β, at each time point, were identified as: mitochondrial precursor Hsp70 (spot 5) in 1 minute; beta-tubulin (spot 9) in 5 minutes; IgE-dependent histamine-releasing factor (IgE-HRF, spot 34) in 30 minutes and glycosomal malate dehydrogenase (GMD, spot 41) in 60 minutes. Proteins like prostaglandin F2-alpha synthase and enolase were identified in two phosphoprotein spots of similar molecular mass, suggesting that these proteins have at least two different sites of phosphorylation. Phosphoprotein spots that showed the highest phosphorylation index at each studied time point are shown in selected zoom areas of images from gels stained with Pro-Q Diamond containing the non-treated and TGF-β treated parasites (Figure 3A and B).

On the other hand, 8 protein spots had their phosphorylation reduced in response to TGF-β addition. The most dephosphorylated proteins observed in each time point of the study were identified as: glycosomal malate dehydrogenase (GMD, spot 41) in 1 minute; prostaglandin F2- alpha synthase (Pf2αS, spot 21) in 5 minutes; hypothetical protein (spot 24) in 30 minutes and mitochondrial precursor Hsp70 (spot 5) in 60 minutes of TGF-β treatment (Figure 3C and D and Table 2).

### Protein Expression Changes in Response to TGF-β

From all 41 identified protein spots showing expression changes in response to TGF-β treatment (Table 3), 5 spots were surprisingly up-regulated already in the first minute of incubation, with cyclophilin A (spot 42) and DnaK (spot 1), two heat shock proteins, showing the highest induction change. At 5 minutes of treatment, 19 protein spots had suffered an increase in expression levels, including the main protein regulated by TGF-β, cruzipain (spot 15), with a dramatic mean 38 fold increase in expression in parasites treated with TGF-β compared to those untreated with this cytokine. In the longer incubation times, 6 protein spots were up-regulated by TGF-β and the main effect was in a hypothetical protein (spot 24) in 30 minutes and tryparedoxin peroxidase (TRYP, spot 38) in 60 minutes of TGF-β stimulation (Figure 4A and B).

Our data also demonstrated that incubation with TGF-β led to a down–regulation in expression of many proteins in the studied time frame. From the 23 protein spots down-regulated by TGF-β, 30% were already down-regulated in the first minute, with Prostaglandin F2- alpha synthase (Pf 2αS, spot 12) being the most repressed. Methylthioadenosine phosphorylase (MTAP, spot 32) was the most down-regulated protein after 5 minutes of TGF-β addition. In the longer incubation times, 20 protein spots had their expression level decreased, with enolase (spot 17) and the chaperonin Hsp70 (spot 3) showing the greatest decrease in expression after 30 and 60 minutes of TGF-β incubation, respectively (Figure 4C and D and Table 3).

Separation of these proteins into functional groups (Figure 5) shows that most of them are involved in metabolic processes (39%), while others are related to heat shock response (24,4%), translation (12,2%), proteolysis (7,3%), cytoskeleton composition (4,9%), signal transduction (4,9%), oxidative stress regulation (2,4%) and hypothetical proteins (7,3%).

Proteins that presented changes in phosphorylation were also separated according to their functional classification (Figure 5, in green). These proteins are involved in metabolism (40%), heat shock response (25%), cytoskeleton (10%), translation (10%), signal transduction (5%), proteolysis (5%) and hypothetical proteins (10%).

Western blot (WB) assays were performed for some proteins (elongation factor 1–alpha, beta-tubulin and cruzipain) shown to be regulated by TGF-β in the proteomic analysis, corroborating the 2-DE results, as observed in Figure 6. Both approaches presented the same kinetic patterns, but the absolute fold increase or decrease values were substantially different, possibly reflecting the known differences in sensitivity between these two methods.

The dramatic increase in cruzipain expression was also confirmed by an immunofluorescence assay (Figure 7), that allowed us to visualize its sub- cellular localization on fixed epimastigotes. The results demonstrated a typical labeling of rounded structures localized at the posterior region, indicating a possible compartmentalization in reservosomes, especially on control parasites (Figure 7A and C). After TGF-β addition, the immunofluorescence analysis of cruzipain staining clearly increased and it was possible to observe a more intense labeling in the flagellar pocket region (Figure 7B and D, white arrow). The intensity of cruzipain staining was quantified using the NIH ImageJ software in the microscopic images of labeled parasites (Figure 7C, D and E).

To test if the changes observed in epimastigotes 2DE profiles after TGF-β addition could be involved in biological events of the parasite’s life cycle, epimastigotes were incubated with or without TGF-β (5 ng/ml TGF-β, added daily) for 96 hours. Our results showed that TGF-β addition promoted an increase in epimastigote proliferation in all studied time points. After 24 hours, we observed an 88% increase in growth compared to epimastigotes cultivated in LIT medium only (Figure 8A). After verifying the biological effect of TGF-β on epimastigotes, we investigated if TGF-β could also influence the amastigote (normally intracellular, replicative) forms of *T. cruzi.* For that, we performed an amastigogenesis assay, in which trypomastigotes were submitted to acidic conditions with or without 5 ng/ml TGF-β for 4 hours. We observed that treatment resulted in a higher rate of differentiation of trypomastigotes into amastigotes (36%) compared to trypomastigotes submitted to acidic conditions only (Figure 8B). These data indicate that TGF-β acts as a powerful regulator in different forms of *T. cruzi*, present both in vertebrate and invertebrate hosts.

## Discussion

In recent years, several groups have used a proteomic approach to study *T. cruzi* biology, trying to identify new factors for diagnostics, virulence, infectivity and mainly, drug targets [29], [31], [32], [34], [40], [41]. Signal transduction pathways are highly dynamic protein networks that integrate information from various stimuli. We and others demonstrated previously the involvement of TGF-β in *T. cruzi* life cycle [6], [7], [8], [12], but a study of parasite proteins responsive to TGF-β stimulus was still lacking. Here, we have profiled for the first time a global and kinetic view of *T. cruzi* epimastigote proteins responsive to TGF-β using a (phospho) proteomic approach.

Reversible protein phosphorylation is a key mechanism for the regulation of major biological processes including metabolism, proliferation, molecule transport and differentiation. Approximately 2% of the *T. cruzi* genome encodes protein kinases [42], suggesting a major regulatory role for protein phosphorylation in parasite development and adaptation.

In this study, we used the Pro-Q Diamond technology, which provides a method for selectively staining phosphoproteins in polyacrylamide gels. Proteomic studies addressing the response triggered by TGF-β in different cellular models have been reported [25], [43], [44], showing that TGF-β stimuli result in negative or positive regulation of proteins involved in diverse functions, such as cytoskeleton arrangement, RNA processing, proteolysis, metabolism and extracellular matrix synthesis. Some of the proteins we found to be regulated by TGF-β have already been described to be directly associated with this molecule, participating in many physiological processes in the life cycle of protozoan parasites.

We observed that the mitochondrial precursor Hsp70 had an 8-fold increase in phosphorylation after one minute of incubation with TGF-β, being one of the proteins to present a fast and strong phosphorylation change in response to TGF-β. Heat shock proteins (Hsps) act as molecular chaperones, binding to a subset of newly synthesized polypeptides to assist their folding to a well-defined three-dimensional conformation [45]. Comparative proteomics of developmental stages of *Leishmania donovani* and *T. cruzi* show differences in expression of Hsp60, Hsp70, mitochondrial Hsp70 and Hsp90 [28], [46]. A recent study [47] reported that Hsp70 interacts with Smad2 and decreases TGF-β signal transduction.

Tryparedoxin peroxidase (TRYP) was also shown to be positively regulated by TGF-β. This protein is described as participating in distinct functions, including general cell detoxification and specific signaling during proliferation or differentiation processes [48]. Our findings lead to the speculation that TGF-β participates in the differentiation process triggered by TRYP corroborating to previous study in which it was observed that the inhibition of TGF-β pathway impaired *T. cruzi* differentiation into trypomastigotes at the end of intracellular cycle [8].

Most of TGF-β-responsive proteins pointed out in our study are associated with metabolic functions. Our data show that phosphorylation of glycosomal malate dehydrogenase (GMD) increases on average 24-fold, 60 minutes after TGF-β addition. This protein participates in sustaining energy supply by acting in parasite’s aromatic amino acid catabolism. Trypanosomatids depend on these nutrients for a number of vital cell functions such as protein synthesis, osmoregulation, energy production and polyamine biosynthesis, since carbohydrates are rarely available in the gut of haematophagous insects [49]. Some proteins, like enolase, presented a reduction in expression levels only in the longest period of induction with TGF-β. Enolase (2-phospho-D-glycerate hydrolase) participates in both glycolysis and gluconeogenesis [50]. It was also described as a prokaryotic RNA degradosome component [51], [52], which might have implications on RNA stabilization and/or degradation, favoring the post-transcriptional control of *T. cruzi* gene expression.

TGF-β also appears to influence the parasite’s cytoskeleton arrangement, through the modulation of β-tubulins, the proteins that form microtubules, and play an important role in the morphological changes associated with the life cycle of trypanosomes [53], [54], [55]. Our data show that this protein is positively regulated by TGF-β, consistent with the role of TGF-β on proliferation and differentiation of the parasite. Tubulin has previously been shown to be up-regulated in hepatic stellate cells treated with TGF-β [56]. Moreover, the association of endogenous Smads with microtubules has been shown to be an important feature of TGF-β signaling [57].

Interestingly, a tubulin binding protein, named IgE-dependent histamine-releasing factor, had its phosphorylation increased 30 minutes after TGF-β stimulus. It is also known as translationally-controlled-tumor-protein (TCTP) and various cellular functions and molecular interactions have been ascribed to this protein, many related to its growth-promoting and anti-apoptotic properties. Since it has been characterized as an anti-apoptotic protein [58], [59] and the blockage of the TGF-β intracellular pathway induces higher levels of intracellular amastigote apoptosis [8], this protein could play a role in *T. cruzi* survival, through the abrogation of apoptosis induction, but further studies should be done to confirm this hypothesis.

In our study, the protein with the highest positive regulation was cruzipain, the major *T. cruzi* papain-like cysteine proteinase. This molecule is expressed in all life-cycle stages of the parasite, being more abundant in replicating forms and especially in the insect epimastigote stage. Cruzipain participates in the nutrition of the parasite at the expense of the host, being the major proteinase of the large acidic prelysosomal compartment called reservosomes. Cruzipain also plays a role in the invasion of host cells by *T. cruzi* trypomastigotes and participates in the escape mechanisms used by the parasite to evade the immune system of the host [60], [61]. Cruzipain has been shown to enhance IL-10 and TGF-β production, favoring parasite survival in macrophages [62]. The involvement of this protein in the capacity of *T. cruzi* to activate TGF-β has already been proposed by us [8] and is under further investigation in our laboratory. Additionally, our results showed that TGF-β was also capable to stimulate *in vitro* amastigogenesis, extending previous data which demonstrate that epimastigotes overexpressing cruzipain have a higher rate of differentiation into metacyclic forms [63].

The absence of classical signaling molecules found in our study may be explained by the low abundance of these proteins, or due to stoichiometry and kinetic factors of their phosphorylation, being considered as a limitation of the chosen technique. However, recently, combination of phosphopeptide enrichment approaches with more sensitive mass spectrometry-based methodologies [64], [65] have been applied to the study of general protein phosphorylation in *T. cruzi* at the epimastigote stage, enabling the identification, quantification and description of site-specific phosphorylation in this microorganism, but failing to identify any of the classical TGF-β signaling molecules. Another hypothesis suggests that TGF-β triggers alternative signaling routes in *T. cruzi,* independent of the pathways already described in more complex eukaryotes.

The idea of a signaling pathway triggered by TGF-β suggests the presence of TGF-β receptor(s) on the *T. cruzi* cell surface in order to initiate an intracellular cascade. However, no orthologs and/or analogs of the canonical serine-threonine kinase TGF-β receptors (TRI and TRII) have been found after *in silico* analysis of the *T. cruzi* genome [12]. A molecular analysis of the probable *T. cruzi* TGF-β receptor protein is under investigation in our laboratory. It should be remarked that other mammalian growth factors (namely epidermal growth factor and TGF-α) have been shown to bind to an EGF-like receptor and induce signal transduction events and cellular proliferation in *T. cruzi* axenic amastigotes [66], [67].

Protein phosphorylation is strongly associated with the processes of cell signaling and development in eukaryotes. Post-transcriptional modifications appear to be even more important for the regulation of gene expression in the Kinetoplastids, since they lack several transcriptional control mechanisms [36], [68], [69]. Besides the quantity of information generated by a phosphoproteomic approach, it is extremely challenging to connect the kinases to their molecular targets. This challenge reflects both the complexity and variety of functions played by these proteins and also the fact that kinases usually exert their biological effects through the simultaneous phosphorylation of different sites of a protein or in multiple protein complexes. We have identified a series of proteins responsive to TGF-β, but the interactions established among them and their role in the signaling triggered by TGF-β remains to be fully understood.

Although we could not determine a linked network between the modulated proteins, the stimulation of epimastigote growth by TGF-β may be partially attributed to positive regulation of proteins like TRYP, tubulin, and TCTP, which have already been described to participate in the process of proliferation and prevention of apoptosis. These functions are compatible with epimastigote biology in the bug’s crop following a blood meal, thus suggesting that TGF-β may play an important role in these processes. In malaria, another parasitological disease, TGF-β appears to have an essential role in the development of insect immunity. It has been reported [70] that during blood meal, the Anopheles mosquito vector ingests a variety of mammalian signaling factors - including TGF-β - that can communicate with immunological cells of the invertebrate host. This study demonstrates that cytokine transmission is not only critical for Plasmodium development in the vertebrate host, but can also influence parasite development in the mosquito, thus indicating that through a conserved immunological cross talk, mammalian and insect immune systems interact with each other to influence the Plasmodium life cycle.

In view of the important role of TGF-β during the parasite life cycle and in development of infection, the present study contributes for the elucidation of *T. cruzi* epimastigote proteins that may be involved in many biological processes in which TGF-β participates, such as invasion, proliferation, differentiation and survival, thereby reinforcing the importance of this molecule in the different forms and stages of the *T. cruzi* life cycle.
